# Evolution of Ag nanostructures created from thin films: UV–vis absorption and its theoretical predictions

**DOI:** 10.3762/bjnano.11.40

**Published:** 2020-03-25

**Authors:** Robert Kozioł, Marcin Łapiński, Paweł Syty, Damian Koszelow, Wojciech Sadowski, Józef E Sienkiewicz, Barbara Kościelska

**Affiliations:** 1 Faculty of Applied Physics and Mathematics, Department of Solid State Physics, Gdansk University of Technology, Gabriela Narutowicza 11/12, 80-233 Gdansk, Poland; 2 Faculty of Applied Physics and Mathematics, Department of Theoretical Physics and Quantum Information, Gdansk University of Technology, Gabriela Narutowicza 11/12, 80-233 Gdansk, Poland; 3 Faculty of Electronics, Telecommunication and Informatics, Department of Biomedical Engineering, Gdansk University of Technology, Gabriela Narutowicza 11/12, 80-233 Gdansk, Poland

**Keywords:** dewetting, finite-difference time-domain (FDTD) method, plasmon resonance, silver (Ag) nanostructures, thin films, UV–vis absorption

## Abstract

Ag-based plasmonic nanostructures were manufactured by thermal annealing of thin metallic films. Structure and morphology were studied using scanning electron microscopy (SEM), transmission electron microscopy (TEM), high-resolution transmission electron microscopy (HR-TEM) and X-ray photoelectron spectroscopy (XPS). SEM images show that the formation of nanostructures is influenced by the initial layer thickness as well as the temperature and the time of annealing. The Ag 3d and Ag 4d XPS spectra are characteristic of nanostructures. The quality of the nanostructures, in terms of their use as plasmonic platforms, is reflected in the UV–vis absorption spectra. The absorption spectrum is dominated by a maximum in the range of 450–500 nm associated with the plasmon resonance. As the initial layer thickness increases, an additional peak appears around 350 nm, which probably corresponds to the quadrupole resonance. For calculations leading to a better illustration of absorption, scattering and overall absorption of light in Ag nanoparticles, the Mie theory is employed. Absorbance and the distribution of the electromagnetic field around the nanostructures are calculated by finite-difference time-domain (FDTD) simulations. For calculations a novel approach based on modelling the whole sample with a realistic shape of the nanoparticles, instead of full spheres, was used. This led to a very good agreement with the experiment.

## Introduction

In the last decade there has been significant development in sensor-related research regarding the application in optical, medical or biological areas [1–5]. The principle of some of these sensors is the resonant enhancement of a local electromagnetic field as well as a sharp spectral absorption, which can be achieved by exploiting localized surface plasmon resonance (LSPR). This phenomenon is based on collective oscillations of free electrons excited by the electromagnetic field of light. The conditions for its occurrence are primarily met by materials with a large number of free electrons, which leads to intensive plasmon resonance and a negative real permittivity over a wide frequency range. Particularly important are noble-metal nanostructures, in which LSPR occurs in the visible spectrum. The frequency of LSPR depends on the size and shape of the nanostructures and the dielectric function of the surrounding medium [6–8]. Regarding a potential implementation, Ag nanoparticles are especially interesting because of their very high extinction cross section. It can be up to 50 times larger than the geometrical cross section of the nanoparticle [6]. Ag nanoparticles are also interesting because of the position of the plasmon resonance. The LSPR wavelength maximum of small Ag nanoparticles with a diameter of 10 nm in air is around 420 nm, which allows for a number of additional applications in comparison with similar Au nanoparticles, which have a maximum at around 530 nm [6]. The resonance position is influenced, for example, by size and shape of the nanoparticles and the surrounding medium. This gives the ability to control the resonance over a wide frequency range. There are many methods for the fabrication of metal nanostructures. One promising technique is the heating of thin metallic films deposited on a substrate. These layers are metastable and can undergo dewetting with increasing temperature. Dewetting can occur via three different processes [9]. In two of them, the formation of nanostructures begins with the nucleation of holes and their subsequent growth. Nucleation can be caused by small thermal density fluctuations (homogeneous nucleation) or defects in the metal film or in the interface between the film and the substrate (heterogeneous nucleation). In the third process, voids appear and grow as a result of an amplification of periodical fluctuations of the film thickness, which is known as spinodal dewetting [9–10]. Usually, it is difficult to state clearly whether only one of the abovementioned processes is responsible for dewetting. Especially because defects are always present in the interface between the substrate and the film. In the design of metallic nanostructure systems for plasmonic applications, the homogeneous distribution of nanostructures is very important, both in size and location on the surface. However, in the case of very simple production methods, as wet chemical synthesis or dewetting, the size of the nanoparticles follows a Gaussian distribution. This work focusses on Ag-based plasmonic platforms manufactured by thermal annealing of thin metallic films. The experimental results are corroborated by FDTD calculations showing the distribution of the electromagnetic field around the Ag nanoparticles, as well as the calculated absorbance. Analytical solutions of the electromagnetic field distribution in plasmonic platforms are known for very simple nanoparticles with spherical or cylindrical shapes. Here, for the first time, a realistic shape of the nanoparticles (according to the TEM images) has been taken into account in the FDTD simulations, instead of modelling them straightforwardly as spheres. Probably the most common example in which the amplification of the local electromagnetic field is extremely important is surface-enhanced Raman scattering (SERS), where Raman spectra can be enhanced by several orders of magnitude. However, there are many other areas where it is possible to increase the efficiency of equipment by increasing the electromagnetic field around metal nanoparticles. For instance, Ag nanoparticles can be used successfully in light emitting diodes, solar cells and photodetectors [11–13]. That is why understanding the relationship between the size and shape of nanostructures and the distribution of the electromagnetic field around the structures is particularly important in the design and optimization of devices based on the plasmon effect.

## Experimental

Ag nanostructures were prepared on borosilicate glass (Corning 1737F) and Si substrates. In both cases, the substrates were cleaned with acetylacetone and then rinsed in ethanol. Thin Ag films (1–9 nm thickness) were deposited using a table-top dc magnetron sputtering coater (EM SCD 500, Leica) in pure Ar plasma (argon, Air Products 99.999%). The Ag target was of 99.99% purity, the rate of layer deposition was about 0.4 nm·s^−1^, and the incident power was in the range of 30–40 W. The layer thickness was measured in situ using a quartz crystal microbalance. To form nanostructures, the as-prepared films were put into a hot furnace and annealed in argon atmosphere at different temperatures for different periods of time. The surface morphology of the samples was analyzed using a FEI Quanta FEG 250 SEM operated at 10 kV. For the analysis of nanograin structure and chemical composition a TALOS F200X HRTEM equipped with an EDS detector was used. SEM and TEM experiments were carried out on samples deposited on silicon substrates.

UV–vis spectra were recorded using a double-beam Thermo Fisher Scientific Evolution 220 spectrophotometer in transmission mode, in a range of 200–1100 nm. For these measurements films were deposited on glass substrates.

The quality of the obtained nanostructures and the valence states of Ag were measured using X-ray photoelectron spectroscopy (XPS, Omicron NanoTechnology spectrometer with 128-channel collector). XPS measurements were performed at room temperature in ultra-high vacuum (ca. 10^−9^ mbar). The photoelectrons were excited by an Mg Kα X-ray source. The X-ray anode was operated at 15 keV and 300 W. An Omicron Argus hemispherical electron analyzer with a round aperture of 4 mm was used for analyzing the emitted photoelectrons. The binding energies were corrected using the background C 1s line (285.0 eV). XPS spectra were analyzed with the Casa-XPS software using a Shirley background subtraction and Gaussian–Lorentzian curves as fitting algorithm.

**Figure 1:**
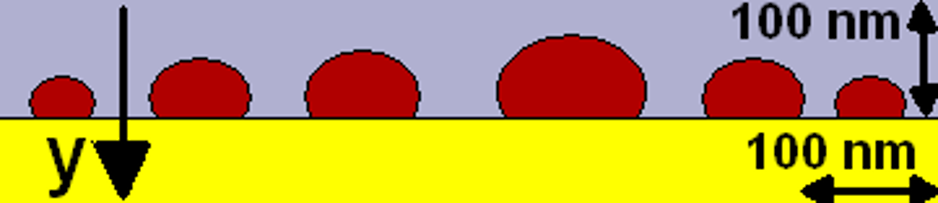
Side view of a part of the simulation setup, illustrating the shape of the Ag nanoparticles, modelled as truncated and flattened spheres (brown) on a Si substrate (yellow), in air (light blue).

**Figure 2:**
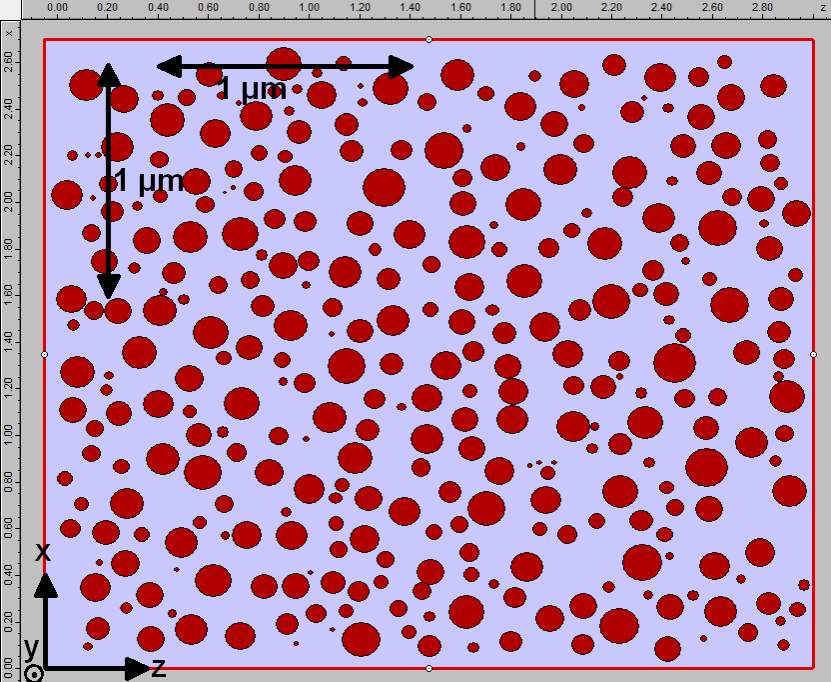
Top view of the simulation setup.

The theoretical three-dimensional simulation of electromagnetic field propagation through a selected sample (thickness 7 nm, annealed at 550 °C for 15 min) was performed using the FDTD method [14], implemented in the OmniSim package, produced by Photon Design, UK. Positions and sizes of the silver nanoparticles, modelled as spheres truncated by 25% and flattened on the *y* axis to 60% of the initial size (Figure 1) on a Si substrate were reproduced on a sample of size 2.7 × 3.0 µm (Figure 2). Modelling the whole sample with regards to a realistic shape of the nanoparticles based on TEM images (see Figure 8 below), instead as full spheres, is a novel approach in the present simulations. It is expected, that this procedure should lead to a better agreement with the experiment.

The grid size for the computations was set to 4 nm (limited by the available computer memory). The dielectric function of silver and silicon was taken from [15] and [16], respectively, and fitted to the Drude–Lorentz model [17] in the range of 287–1000 nm. The fitting error for Si was 1%/0.79% (real/imaginary part), and that for Ag was 0.12%/6.1%. These errors were calculated as the root mean square of the original curves and the fitted ones. Apart from the above, the choice of the FDTD grid size is the main source of error in the present simulations, which is unfortunately rather hard to estimate. However, since the smallest particle in the simulated sample has a diameter of 12 nm, the choice of a three times smaller grid size should keep this error reasonably small. Also, the thin SiO_2_ layer has been neglected, because it is thinner that the computational grid and cannot be correctly modelled. We have performed complementary short simulations with a very limited sample size and with a 2 nm grid, which showed that errors coming from the SiO_2_ layer do not exceed 2%. A light source of 460 nm wavelength has been used, propagating along the *y* axis direction. Two independent simulations were performed, one with a transverse-electric (TE) polarized beam and the second one with a transverse-magnetic (TM) polarized beam. These perpendicularly polarized beams yield, by averaging, the result for the unpolarized beam, according to the formula:

[1] $$\left|E_{\text{unpolarized}}\right|=\sqrt{0.5{\left|E_{\text{TE}}\right|}^{2}+0.5{\left|E_{\text{TM}}\right|}^{2}},$$

where *E*_TE_ and *E*_TM_ are electric and magnetic fields obtained from simulations with TE and TM beam polarizations, respectively. For calculations of the electric field distribution, the shape of the pulse was set to rectangular. The pulse duration was set to 20 fs and the overall simulation time to 100 fs. This was enough to observe plasmonic decay. The field was calculated in a plane located 10 nm above the substrate. For calculations of the absorbance, the pulse shape was set to sinusoidal. The pulse duration was set to 2.5 fs, thus it contained all frequencies within the visible light range. This allowed for the use of discrete Fourier transformation for switching from the time domain to the frequency domain and calculating the spectral response of the sample.

Also, the Mie theory [18] was employed to calculate the scattering efficiencies for a single silver nanoparticle (described by the same dielectric function as previously), surrounded by air. This was done in order to describe absorption, scattering and overall extinction maxima as function of the size of the nanoparticles, since the FDTD simulations are only capable to determine the absorption spectra.

## Results and Discussion

### Structure and UV–vis absorption

**Figure 3:**
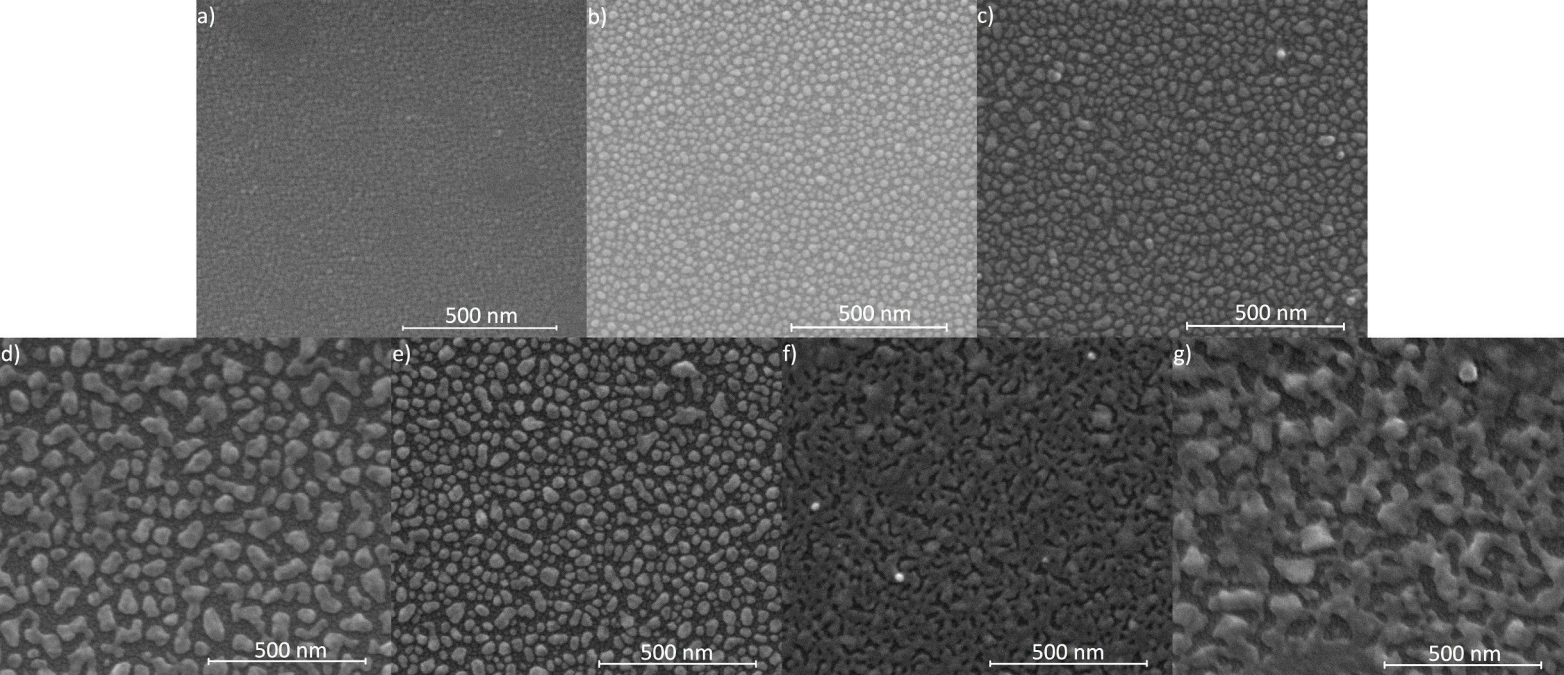
SEM images of the films after annealing at 250 °C for 15 min. Initial film thickness was: (a) 1 nm, (b) 2 nm, (c) 3 nm, (d) 4 nm, (e) 5 nm, (f) 6 nm and (g) 7 nm.

**Figure 4:**
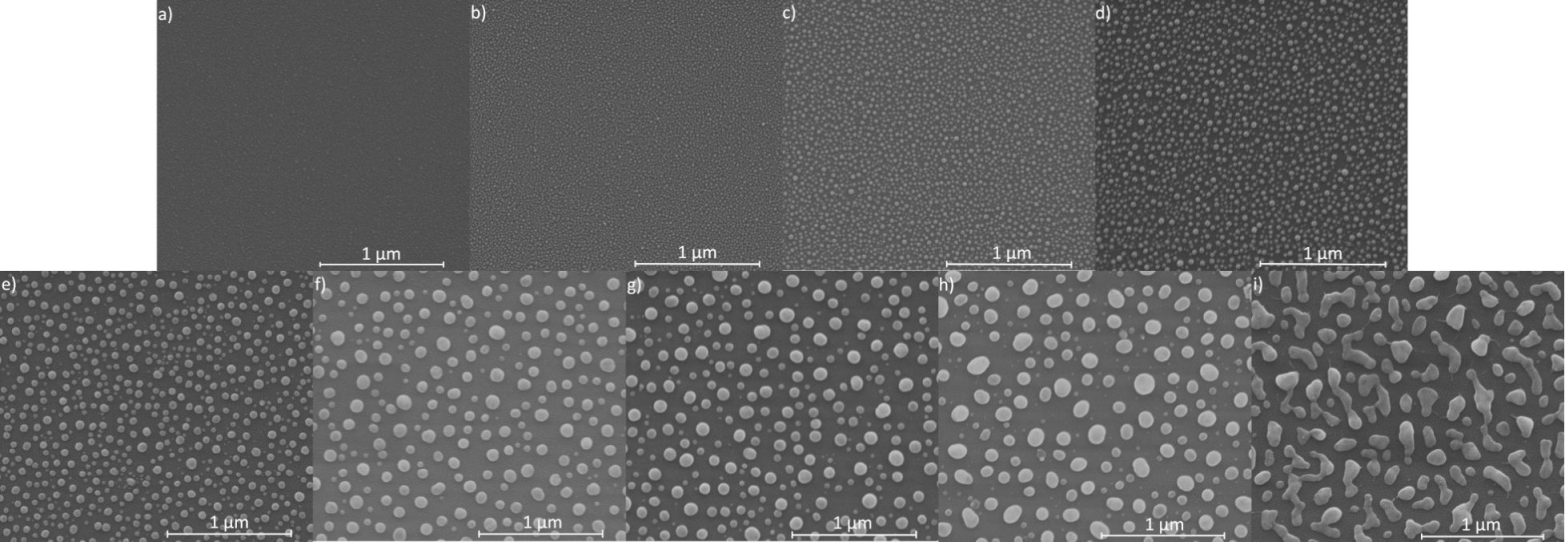
SEM images of the films after annealing at 550 °C for 15 min. Initial film thickness was: (a) 1 nm, (b) 2 nm, (c) 3 nm, (d) 4 nm, (e) 5 nm, (f) 6 nm, (g) 7 nm, (h) 8 nm and (i) 9 nm.

**Figure 5:**
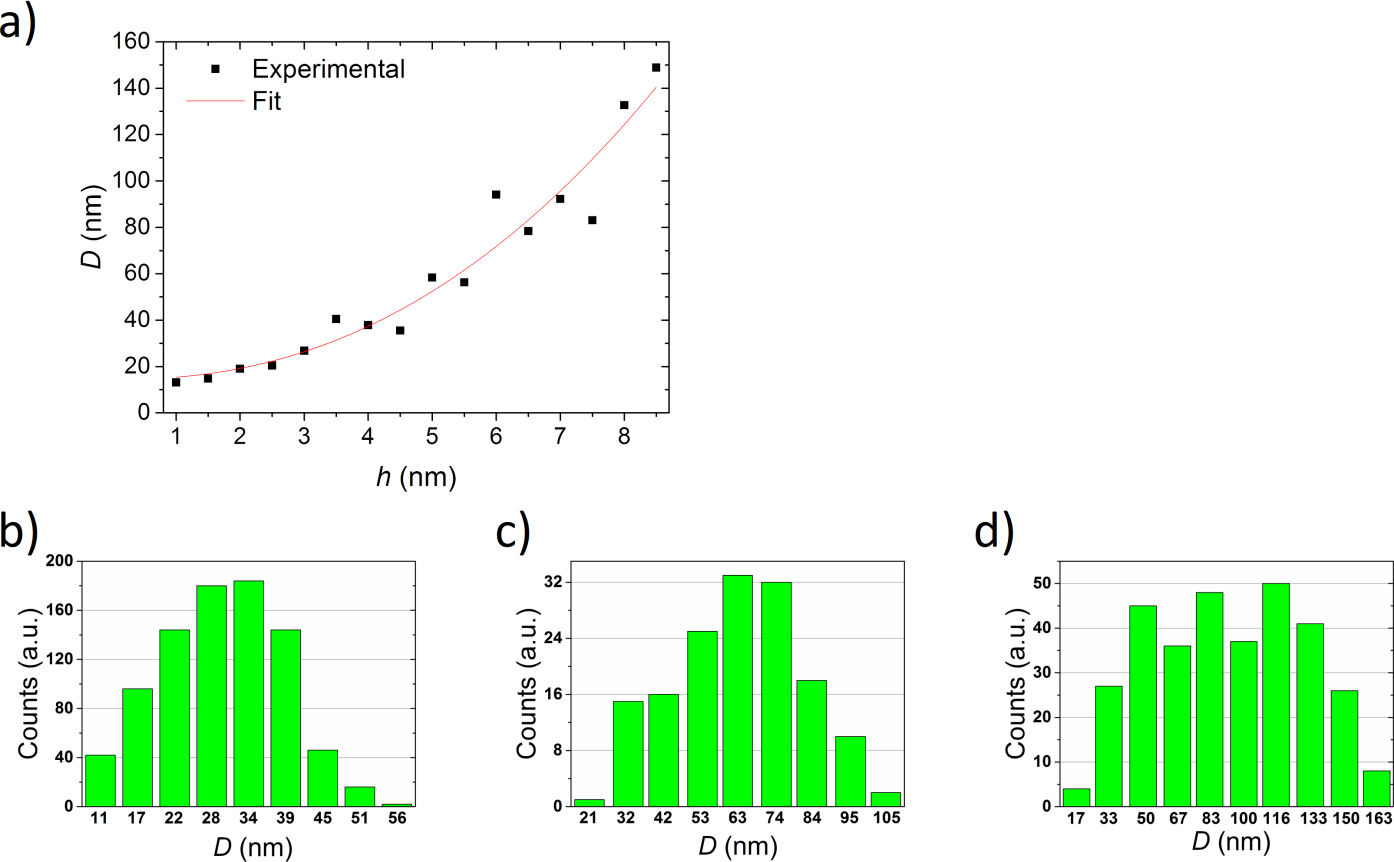
The dependence of the island diameter on the thickness of the initial film, calculated for Ag films annealed at 550 °C for (a) 15 min and the particle size histograms calculated for the nanoparticles obtained after annealing of films with an initial thickness of (b) 3 nm, (c) 5 nm and (d) 7 nm.

The SEM results show that the formation of nanostructures from thin metallic layers is influenced by the initial layer thickness as well as the temperature and the time of annealing. In Figure 3a–g selected SEM images of nanostructures formed after annealing of Ag films with a thickness from 2 to 7 nm are presented. The films were annealed at 250 °C for 15 min. In the image corresponding to the 1 nm thin film (Figure 3a) only some voids are present. There are no islands clearly separated from each other. In the image corresponding to 2 and 3 nm thick layers (Figure 3b,c), clear nanostructures with symmetrical shapes are already visible. The nanoparticles have an average diameter of 37 and 54 nm, respectively. From 4 and 5 nm thick films (Figure 3d,e) no symmetrical nanostructures are formed. However, the nanostructures are still isolated from each other. Separated nanostructures were not obtained from thicker layers. Instead, longitudinal islands and numerous holes and voids are visible (Figure 3f,g). Annealing of the layers at higher temperatures gives much better results. Exemplary SEM images of the films with initial thicknesses from 2 to 9 nm, annealed at 550 °C for 15 min, are shown in Figure 4a–i. Clear nanostructures already appear in this case for a 1 nm thick film. The mean diameter of the nanostructures changes from 13, 19, 27, 38, 58, 94, 92 and 198 nm, respectively, for the films of 1 nm (Figure 4a), 2 nm (Figure 4b), 3 nm (Figure 4c), 4 nm (Figure 4d), 5 nm (Figure 4e), 6 nm (Figure 4f), 7 nm (Figure 4g) and 8 nm (Figure 4h). From the 9 nm thick layer very irregular structures are formed (Figure 4i), although it is probable that an increase in the annealing time could affect their shape. As the thickness of the layer increases, the mean diameter of the nanostructures also increases, but their number decreases. After obtaining the mean nanoparticle diameter (*D*), the mean spacing between them (*s*) and the initial film thickness (*h*), it is possible to specify the type of dewetting [19–21]. If dewetting is of the spinodal type, then the above parameters are related in the following way:

[2] $$D={\left(\frac{24\pi^{3}\gamma }{Af\left(\theta \right)}\right)}^{1/3}h^{5/3}=Ch^{5/3},$$

where *f*(θ) is a geometric factor based on the particle contact angle θ, γ is the surface tension of the metal and *A* is the Hamaker constant. This is valid for the temperature at which isolated islands begin to appear. In this work, however, we focused on temperatures at which we do not only observe isolated islands. These islands also need to be uniform in size and shape. The exponent calculated then differs significantly from the value typical for spinodal dewetting. The exponent calculated for the films annealed at 550 °C for 15 min is 2.26 ± 0.03. The dependence of the diameter of the islands on the thickness of the initial film, on the basis of which the calculations were made, is presented in Figure 5a. Figure 5b–d presents the particle size histograms calculated for the nanoparticles obtained from an initially 3, 5 and 7 nm thick layer, respectively, after annealing at 550 °C for 15 min. In this case, however, it is possible that the edges of the nanostructures have already slightly melted. The authors of this work observed a similar exponent for Au nanostructures obtained by the same method [22].

**Figure 6:**
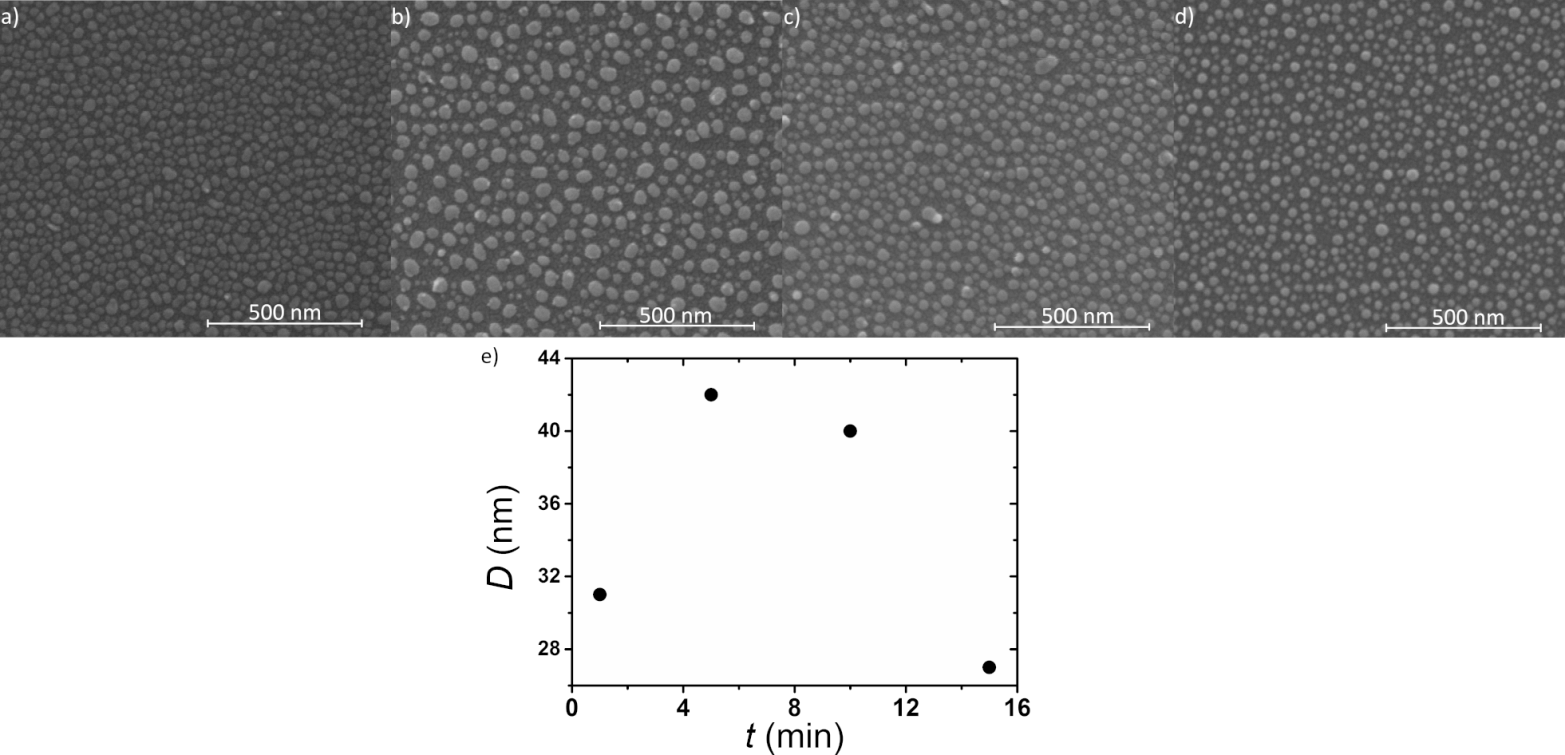
SEM images of a 2.8 nm thick film, annealed at a constant temperature of 550 °C for different periods of time: (a) 1 min, (b) 5 min, (c) 10 min and (d) 15 min; (e) average nanostructure diameter as a function of the annealing time.

**Figure 7:**
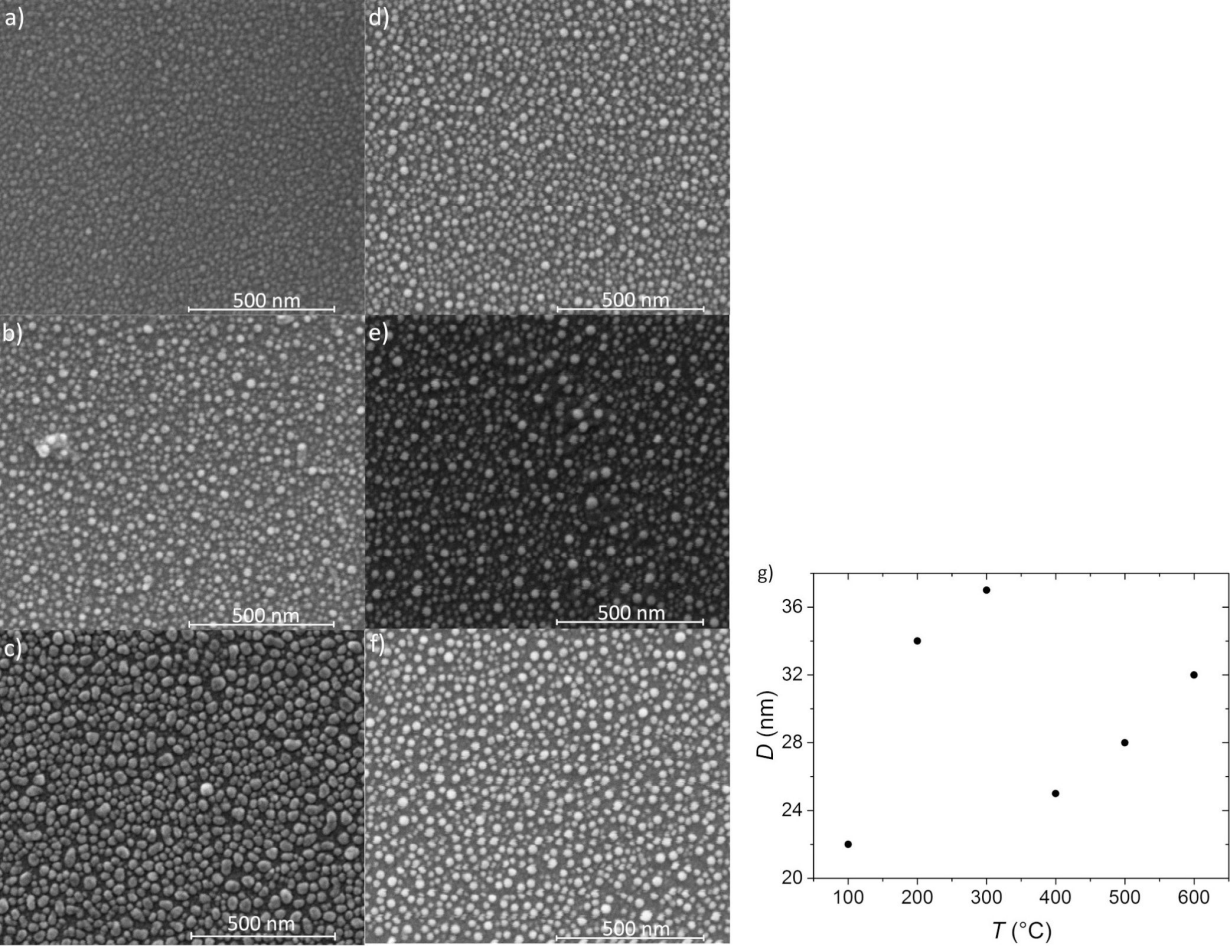
SEM images of a 2.8 nm thick film, annealed for a constant time 15 min at different temperatures: (a) 100 °C, (b) 200 °C, (c) 300 °C, (d) 400 °C, (e) 500 °C and (f) 600 °C; (g) average nanostructure diameter as a function of the annealing temperature.

The impact of annealing time on the formation of nanostructures heated at a constant temperature of 550 °C can be seen in the SEM images shown in Figure 6a–d. The initial layer thickness was 2.8 nm, the annealing time varied from 1 to 15 min. The average diameter of the nanostructures is 31, 42, 40 and 27, respectively, after 1 min (Figure 6a), 5 min (Figure 6b), 10 min (Figure 6c) and 15 min (Figure 6d) of annealing. Hence, the size of the nanostructures does not increase with annealing time. The influence of the annealing temperature on the formation of the nanostructures is shown in Figure 7a–g.

**Figure 8:**
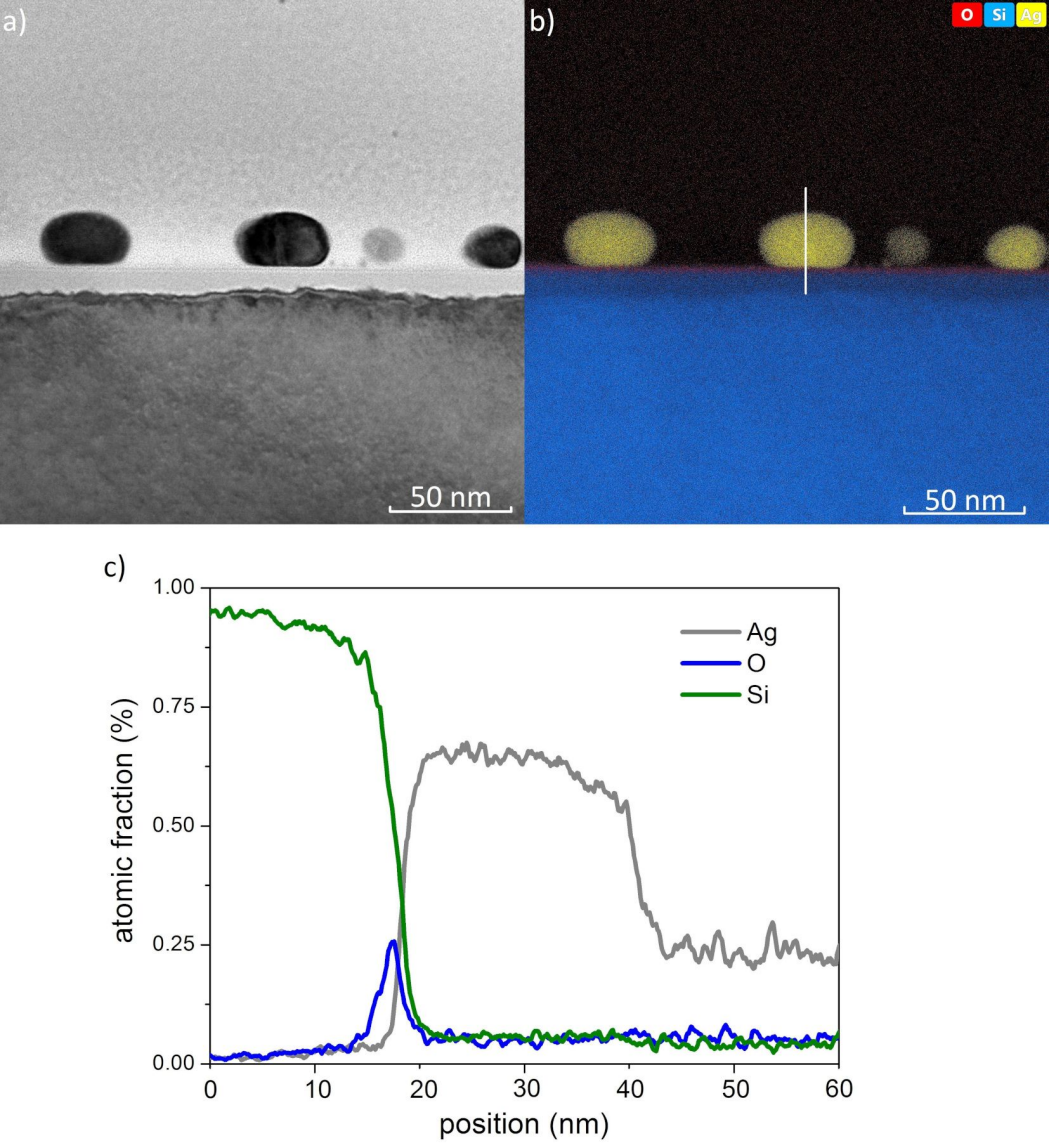
(a) HRTEM image of a cross section of a nanoisland formed from a 3 nm thick film, annealed at 550 °C for 15 min; (b) EDS analysis and (c) detailed EDS analysis of the cross section of the nanoisland.

The shape of nanostructures is clearly visible in the HRTEM image (Figure 8a). The film from which the nanostructures were formed was 3 nm thick and was annealed at 550 °C for 15 min. The nanostructures are slightly flattened, but as follows from EDS analysis (Figure 8b), they consist of Ag. Detailed EDS analysis of a cross section of a nanoisland is presented in Figure 8c. As can be seen, a thin layer of natural SiO_2_, about 2 nm thick, is present on the silicon surface. Interestingly, there is no oxide layer around the Ag nanostructures.

**Figure 9:**
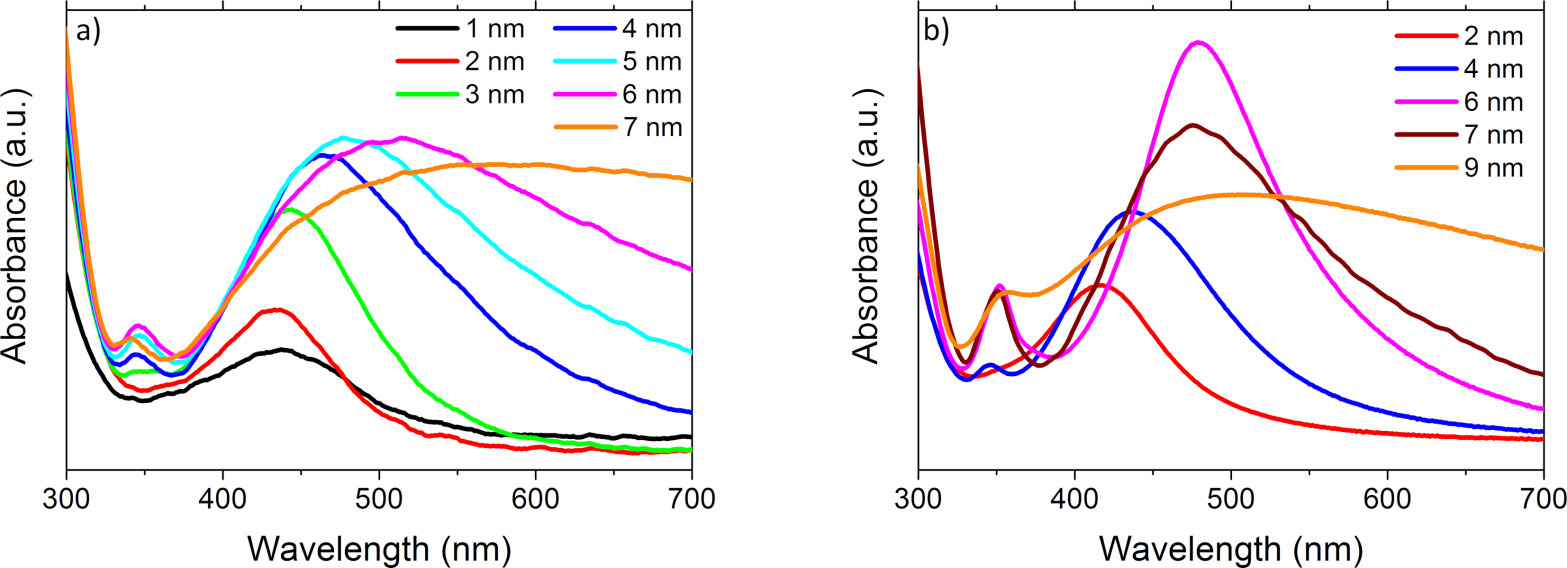
Absorbance of nanostructures formed from thin films with different thickness, annealed at (a) 250 °C and (b) 550 °C for 15 min.

The quality of the nanostructures resulting from the thermal treatment (in view of the presence of plasmon resonance) is reflected in the UV–vis absorption spectra. The UV–vis spectrum of Ag nanostructures is quite complicated. It can be influenced by many factors, such as the size of nanostructures, their shape, changes in the electronic structure, or the dielectric function of the medium in which such nanostructures are dispersed. Probably the simplest case are small nanostructures (*D* ≪ λ) with a spherical shape. Then, the resonance reflects only the dipole mode of the collective oscillations of electrons. Furthermore, if the size of the metallic nanoparticles is larger than 10 nm, their dielectric functions are known to be independent on the size and to have the values of the bulk material [7]. For instance, a dipole mode can then be excited when ε = −2, which corresponds to λ = 355 nm for Ag and λ = 490 nm for Au. When the particle size is increasing, the resonance peak is broadened, its position is red-shifted and additional higher-order resonances can appear. A much more complicated situation occurs in the case of other (e.g., elliptical) shapes of nanostructures. For elliptical nanostructures, two resonances can be observed, corresponding to the illumination in a direction parallel or perpendicular to its major axis [23]. In recent years, many scientific papers have also been devoted to triangular or tetrahedral nanoparticles, or nanorods/nanotubes with complex cross sections [23–25]. Plasmon resonance, and thus the amplification of the electromagnetic field, was observed in several directions, depending on the direction of illumination. The plasmon resonance also has an effect on the porosity of the metallic nanostructures [26–27], which is why determining the shape and surface quality is extremely important. The absorbance of films with a different initial thickness, annealed at 250 and 550 °C is shown in Figure 9a and Figure 9b, respectively. The 1 nm thick film exhibits a broad bump in the range 350–550 nm, which is possibly associated with the occurrence of collective vibrations of free electrons (Figure 9a). This bump becomes more intense and narrower for larger layer thicknesses. For a 6 nm thick layer, it begins to expand and disappear. However, the results obtained from SEM show that annealing of 6 and 7 nm thick films does not lead to the formation of symmetrical isolated nanostructures. In the spectrum corresponding to the 3 nm layer, an additional maximum appears at about 350 nm, which is also present in the spectra of thicker samples.

In metals, valence and conduction bands can overlap, which leads to a continuous spectrum of sites available for electrons. However, for inner levels, it is possible that they will not split sufficiently to provide such an overlap. It should be more evident especially in nanostructures, in which the number of atoms is reduced compared to the bulk material. In this case, under the influence of light of the appropriate wavelength, electrons can exhibit transitions between separated bands. This can be observed in Ag, where a transition of electrons induced by visible light can take place between the d band and the sp band [6–7,28–32]. The width of the gap between s band and d band is in this case in the range of 3.7–3.9 eV [7,29,31], which corresponds to wavelengths in the range of 335–318 nm. Unfortunately, for our samples, no peak can be seen in this wavelength range. Maybe because this is the wavelength range at which absorption is also affected by the substrate. It should be added, however, that in some works weak peaks at other wavelengths were also attributed to interband transitions [30,33].

**Figure 10:**
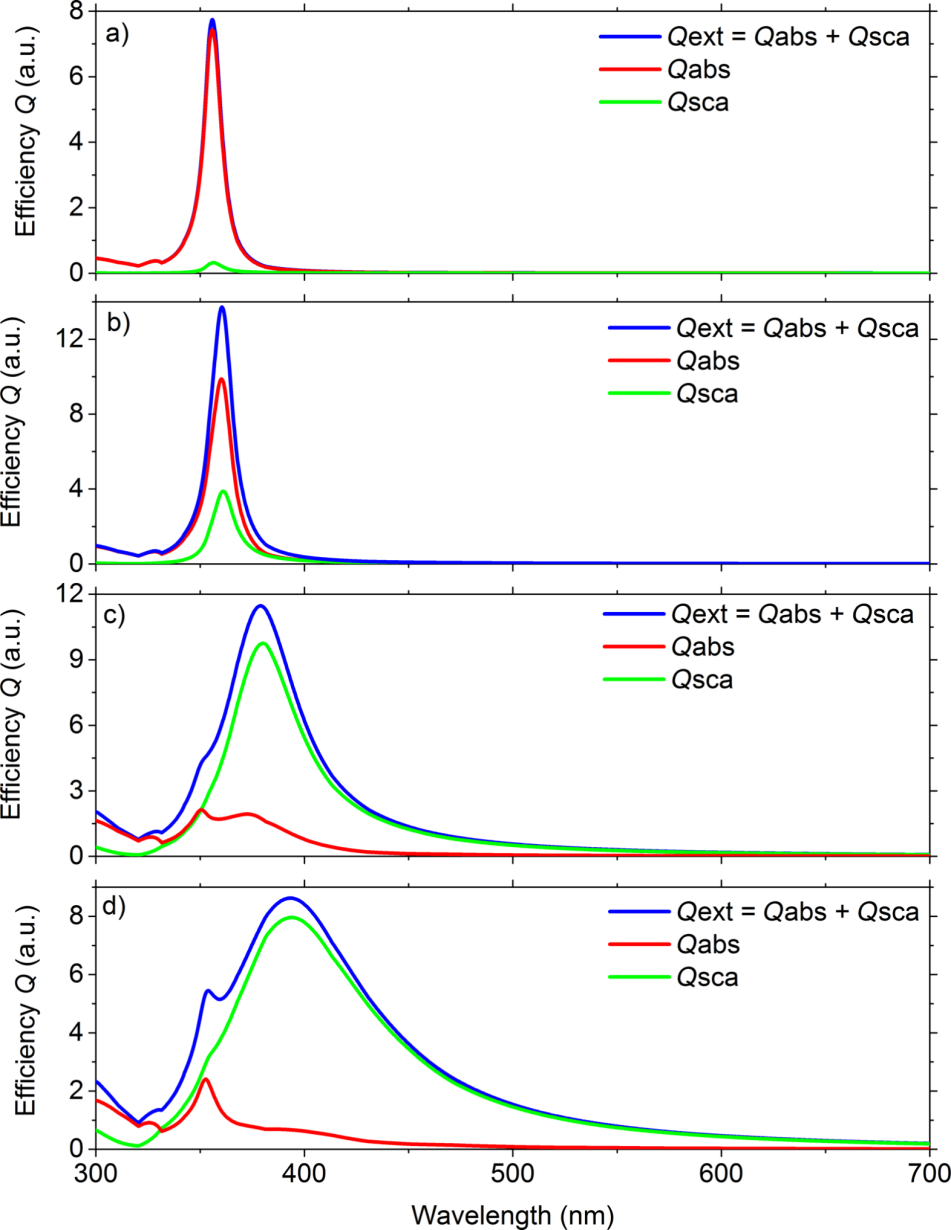
Calculated scattering efficiencies (absorption, scattering and extinction) in air obtained from Mie theory for a single silver nanoparticle with a diameter of: (a) 20 nm, (b) 40 nm, (c) 60 nm and (d) 100 nm.

The peak at the wavelength at about 350–360 nm could correspond to quadrupole resonance [34–39]. Quadrupole resonance is usually observed for nanostructures of larger size, in nanorods with pentagonal cross section, or in hexagonally or pentagonally shaped nanoparticles. If you were to look at the theoretical spectra, calculated directly on the basis of Mie theory (Figure 10a–d), an additional peak (between the peak resulting from interband transitions and the peak from dipole plasmon resonance) appears already for 60 nm diameter nanoparticles. Although the theory does not directly indicate that this is a peak associated with quadrupole resonance. It is also possible to find information that its nature is not fully understood [39–40].

**Figure 11:**
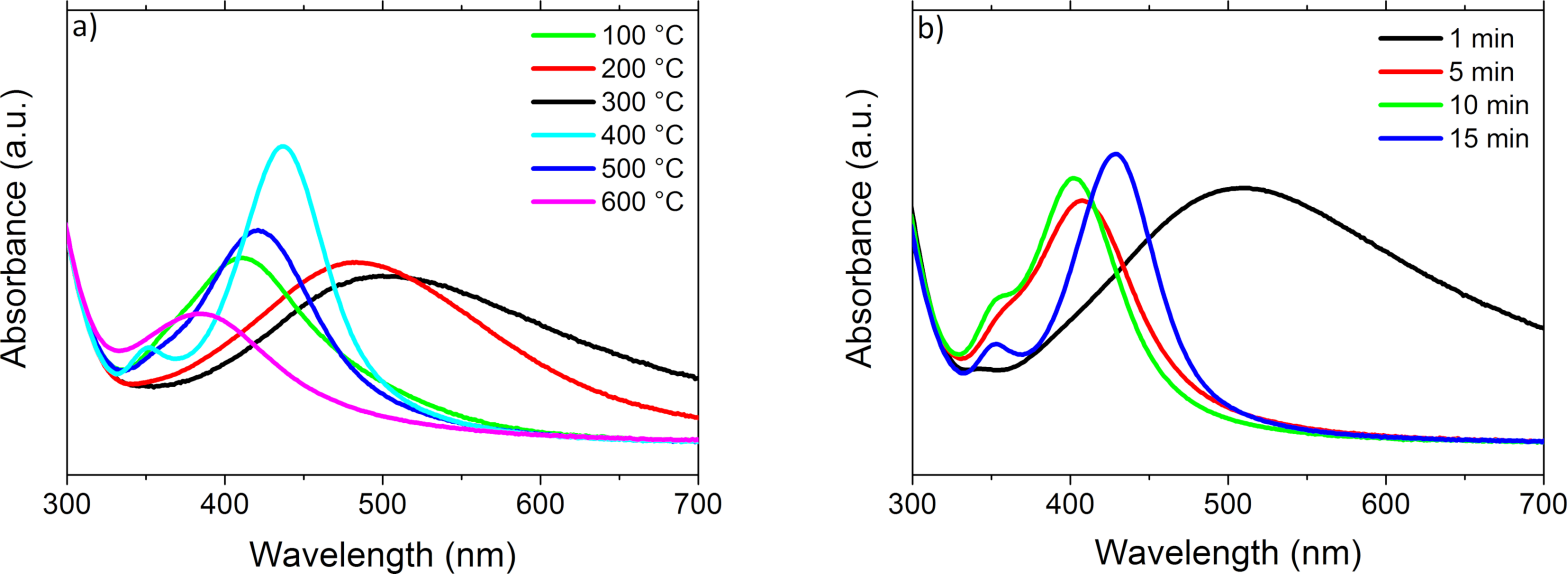
Absorbance of nanostructures formed from 2.8 nm thick films (a) annealed at 100, 200, 300, 400, 500 and 600 °C for 15 min and (b) annealed for 1, 5, 10 and 15 min at 550 °C.

The effect of temperature and annealing time on the absorbance of the nanostructures is shown in Figure 11a and Figure 11b, respectively. The most intense peak resulting from plasmon resonance corresponds to an annealing temperature of 400 °C (Figure 11a). At this temperature a second peak is observed for a wavelength of about 350 nm. In turn, Figure 11b shows that plasmon resonance is significantly affected by the annealing time of the layers. It also affects the appearance of the second maximum, which is related to the size of nanoparticles.

**Figure 12:**
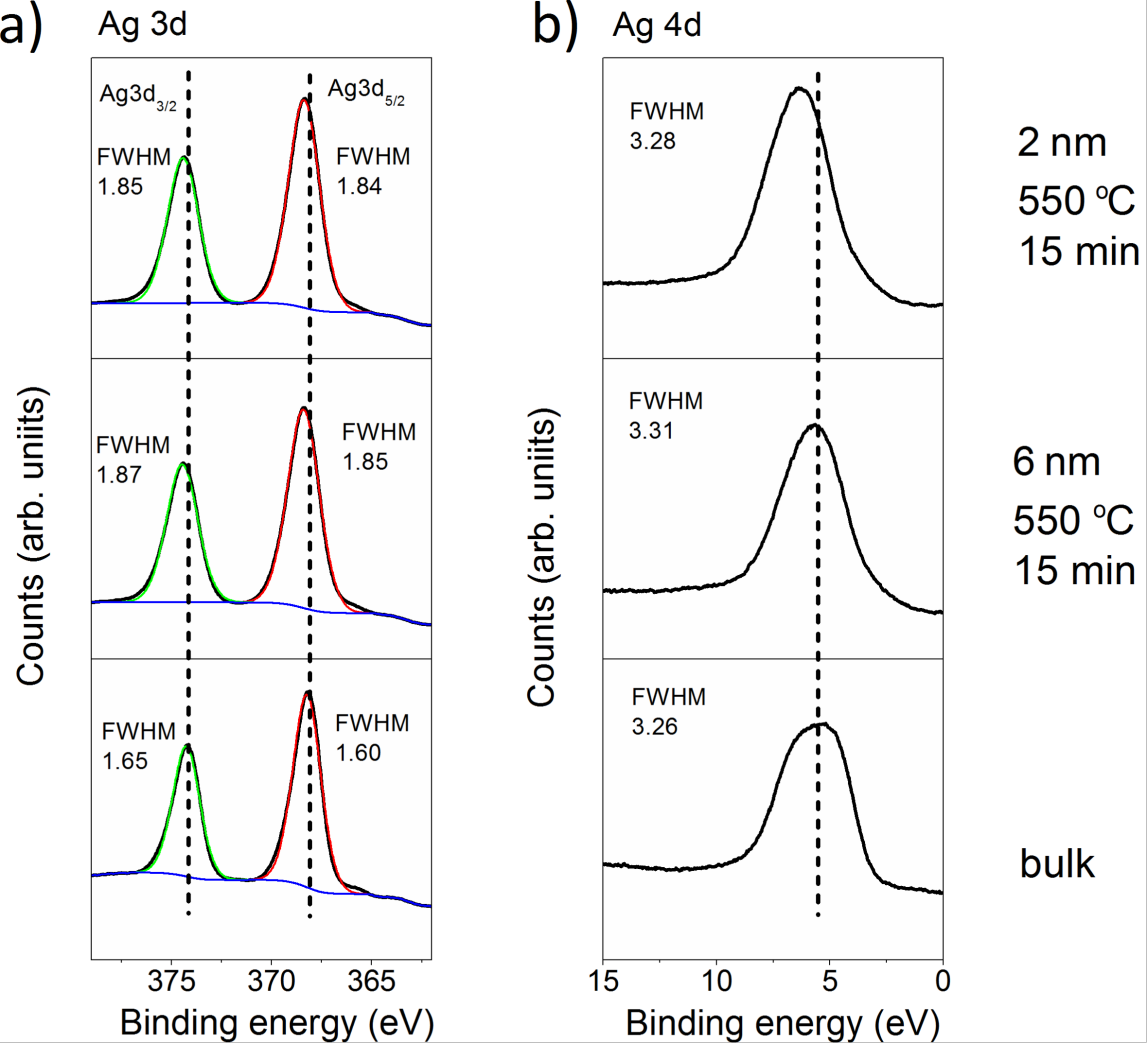
(a) Ag 3d and (b) Ag 4d XPS spectra of bulk Ag and Ag nanostructures grown after annealing of 2 nm and 6 nm thin films at 550 °C for 15 min.

The changes in the electronic structure of Ag caused by the transition from bulk material or thin films to nanostructures can be observed using XPS. The spectral contribution of 5s and 5p electronic states to the valence band spectrum of Ag is negligible and the valence band mainly originates from 4d electronic state. The Ag 3d valence-band and Ag 4d core-level spectra are shown, respectively, in Figure 12a and Figure 12b. It should be noted that no peaks characteristic of Ag–O compounds were observed, which is consistent with the EDS results presented above. Figure 12 presents the results for samples that differ in the UV–vis spectra. For comparison, the spectrum of bulk Ag was added. As it can be seen, a slight shift of the 3d peaks in relation to the bulk material can be observed for both samples, with an initial layer thickness of 2 and 6 nm. Whereas the 4d peak shifts only for the sample with an initial layer thickness of 2 nm. The size-induced change can also be observed when looking at the full width at half maximum (FWHM). FWHM values both of the valence-band and core-level spectra are clearly larger for the nanostructures than for the bulk material. The changes in the peak position originating from the d bands and the broadening of the FWHM can be attributed to the modified electronic structure [33]. In turn, it may be reflected in the UV–vis absorption spectrum. However, in the case of the tested samples, no changes in the UV–vis spectrum associated with the electronic structure were observed.

### FDTD simulation results

**Figure 13:**
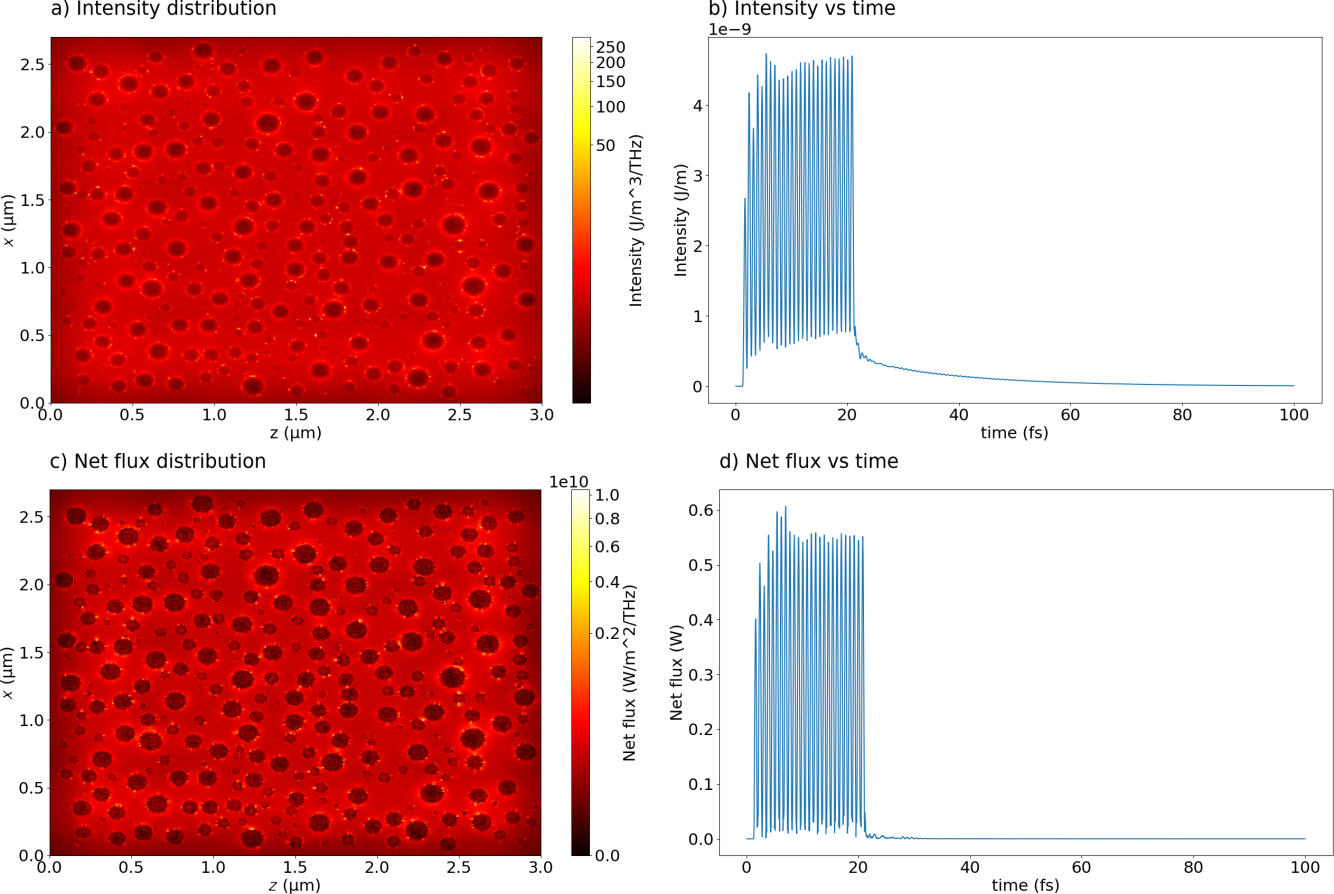
a) Calculated intensity distribution of the electromagnetic field integrated on the plane where nanoparticles are placed; b) intensity amplitude as a function of the time; c) calculated net flux (transmission minus reflection); d) net flux amplitude as a function of the time. The incident light at wavelength 460 nm is unpolarized.

**Figure 14:**
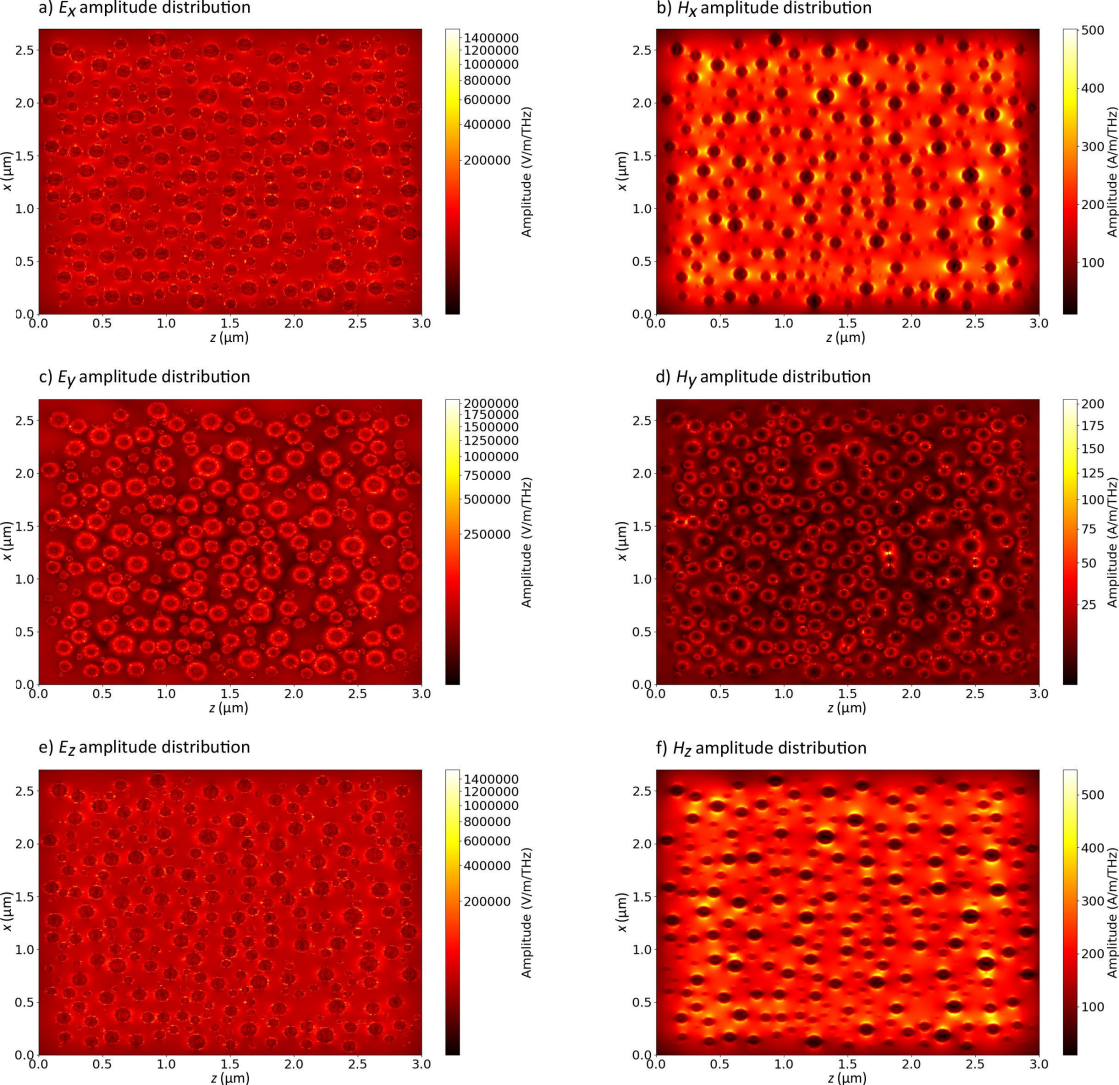
Calculated amplitudes of the components of the electromagnetic field in the plane where the nanoparticles are placed. The incident light at wavelength 460 nm is unpolarized. From (a) to (f): *E_x_*, *H_x_*, *E_y_*, *H_y_*, *E_z_* and *H_z_*.

In Figure 13 the calculated intensity distribution (Figure 13a) and its amplitude as a function of the time (Figure 13b) are presented, as well as the calculated net flux (transmission minus reflection, Figure 13c,d). Since most of the interparticle gaps are not much shorter than the length of the incoming light wave and the nanoparticles sizes, these gaps do not fully support interparticle connections resulting in hot spots for field enhancement. Thus, they only yield a relatively weak enhancement of the scattered field filling almost all gaps on the entire plane. But we can still identify a few interparticle gaps where we observe strong (two to five times) intensity enhancements (hot spots). Also, plasmonic decay is also clearly visible, while the pulse is passing through the nanoparticles. In Figure 14, the amplitudes of all components of the electromagnetic field are presented.

**Figure 15:**
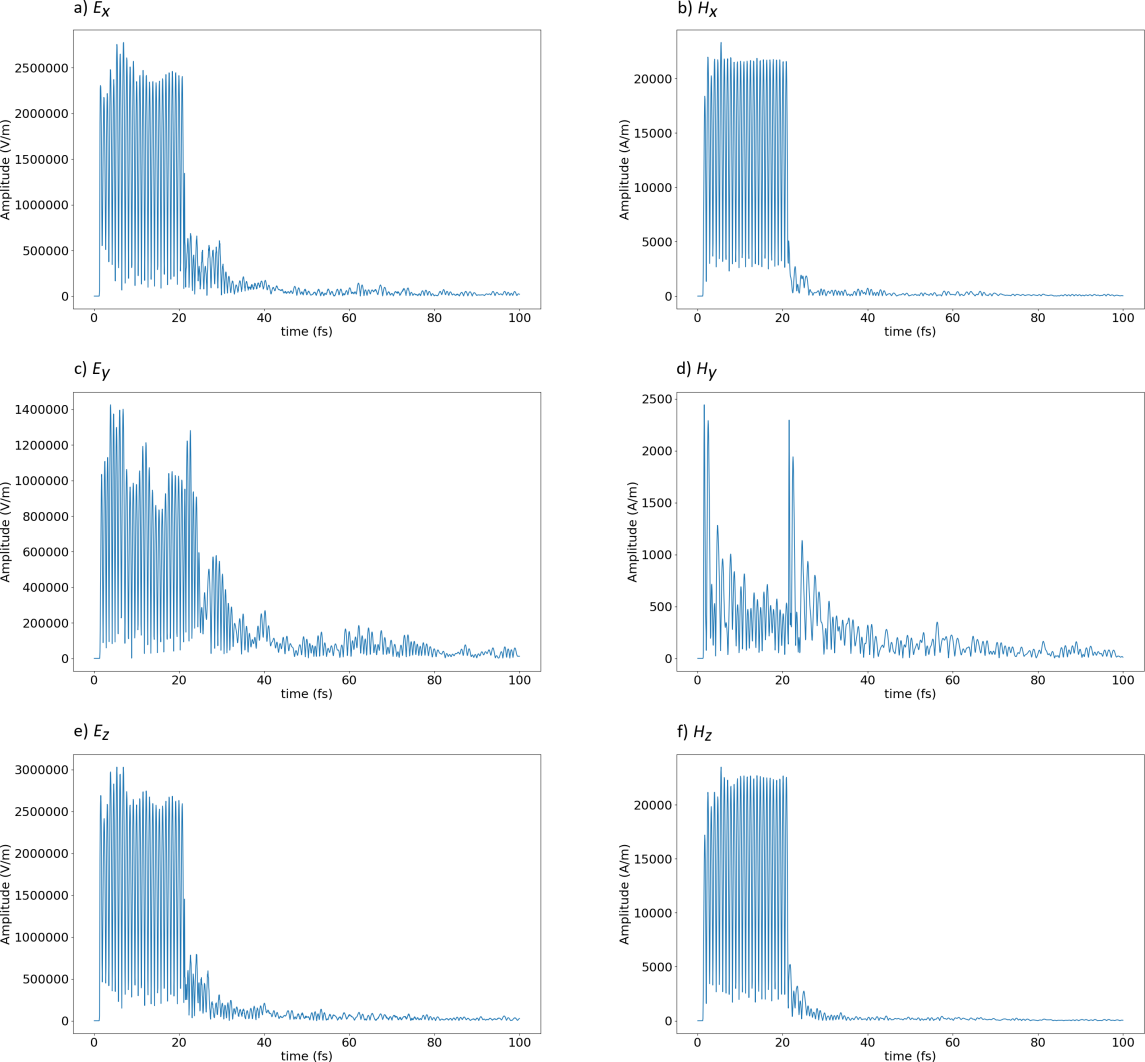
Calculated amplitudes of the components of the electromagnetic field as a function of the time, at the middle of the sample. The incident light at wavelength 460 nm is unpolarized. From (a) to (f): *E_x_*, *H_x_*, *E_y_*, *H_y_*, *E_z_* and *H_z_*.

In Figure 15, amplitudes of the electromagnetic field, in this case measured in the middle of the sample, are plotted as a function of the time. We can observe how these amplitudes increase (sharp rise at approx. 0.9 fs after the start of the simulation), and then decay after the light is switched off and passed the sensor (beginning at approx. 20.9 fs).

**Figure 16:**
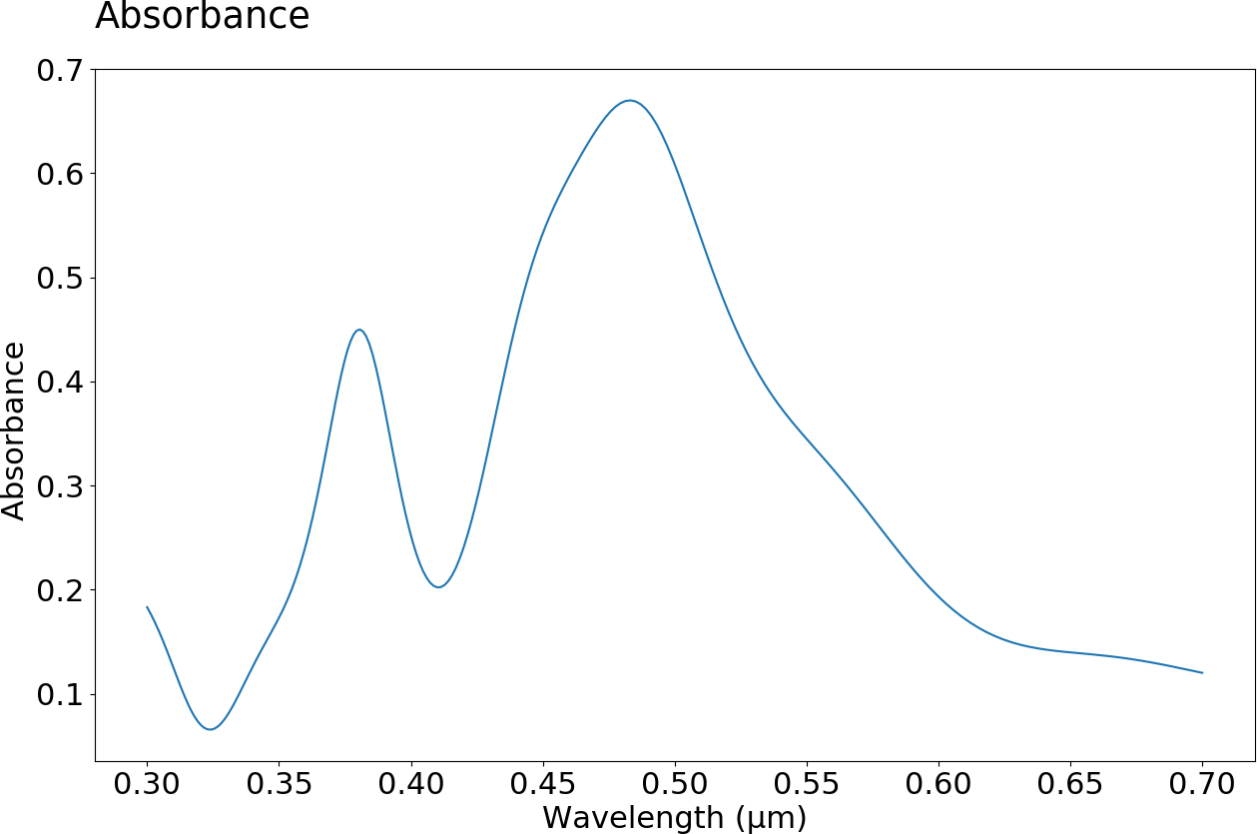
Calculated absorbance log(Φ_i_/Φ_t_), where Φ_i_ and Φ_t_ are incident and transmitted flux, respectively, as the function of the incident light wavelength.

**Figure 17:**
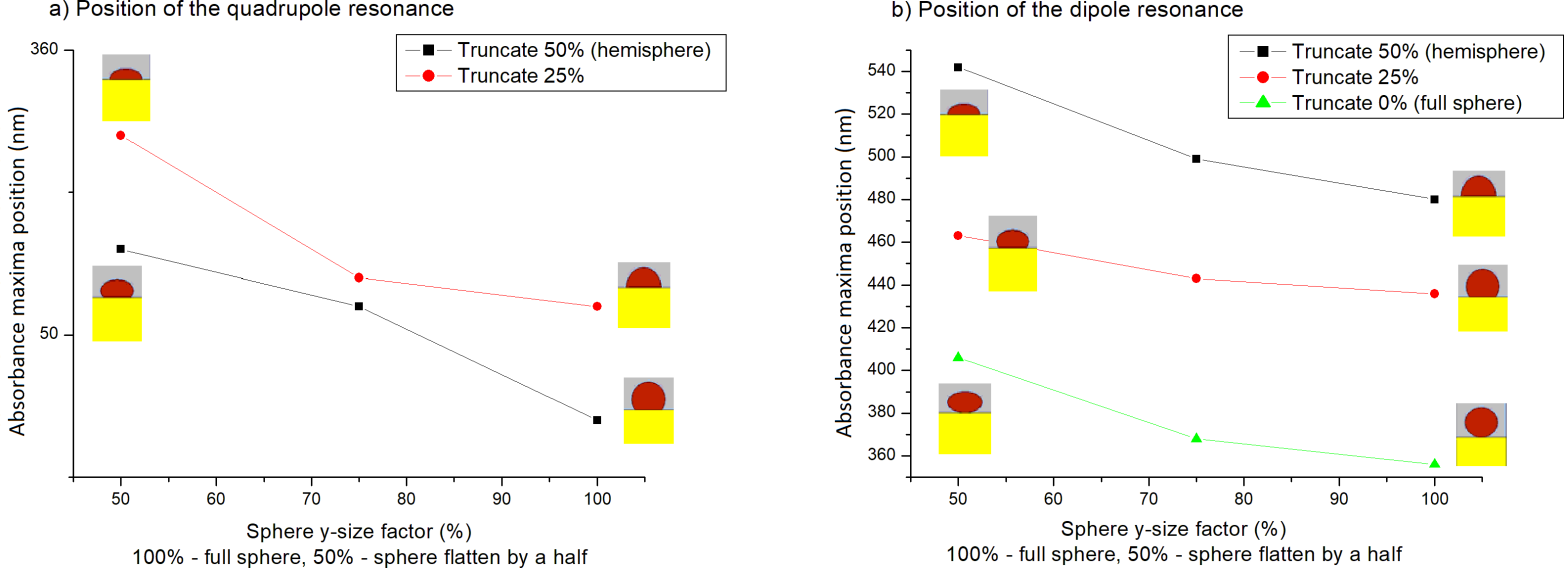
Positions of a) the first and b) the second absorbance maxima (corresponding to the quadrupole and dipole resonance), calculated using FDTD for a single Ag nanoparticle of a size of 100 nm, modeled as a sphere with different truncation and flattening factors on a glass substrate. In the case of a full sphere only dipole resonance occurs.

The absorbance calculated from the FDTD simulations is shown in Figure 16. The two local maxima, corresponding to dipole and quadrupole resonances, are located at 380 and 484 nm, respectively. Generally speaking, the overall agreement with the experiment is quite good. The differences between simulation and experimental data can be explained by the fact that in the simulation all nanoparticles were modeled as truncated and flattened spheres of similar shape. Clearly, truncating and flattening the nanoparticles has an influence on the position of the resonances (especially the dipole resonance), which is illustrated in Figure 17. These resonances were calculated in supplementary FDTD simulations. Also, a FDTD grid of 4 nm was used, which might be not small enough to perfectly reproduce the experimental data (the smallest nanoparticles in the simulated sample were of 12 nm diameter). The grid size is limited by the available memory of the computational cluster used. We plan to use a more advanced and less memory-consuming method, FETD (finite elements in time domain, FEM), in the future.

## Conclusion

This work presents synthesis and UV–vis absorption studies of Ag nanostructures deposited on glass or silicon substrates. As shown, the formation of nanostructures from thin metallic films is influenced by the initial layer thickness as well as the temperature and the time of annealing. In the UV–vis spectra of some samples, apart from the characteristic for plasmon resonance peak, an additional peak with a maximum at 350 nm is visible. It is correlated with the size of the nanoparticles and it is probably the result of quadrupole resonance. Interestingly, no interband transition was observed in the spectra. The shape of the experimental UV–vis spectrum is consistent with the calculations obtained from FDTD calculations. For the calculations, a novel approach based on modelling the whole sample with a realistic shape of the nanoparticles, instead of full spheres, was used. As it can be noticed, the overall agreement with the experiment is quite good. The existing difference between simulation and experimental data can be explained by the fact that in the simulation all nanoparticles were modeled as truncated and flattened spheres with a similar shape. Both truncating and flattening affect the position of the resonances.

A simple method of annealing thin metallic layers leads to the formation of Ag nanostructures with the desired dimensions. The position of the resonance peak and the appearance of quadrupole resonance in the UV–vis spectrum can be accurately predicted using FDTD calculations, which leads to great opportunities in the design of platforms with specific properties, e.g., in nanosensors.
